# Continuous representation of tumor microvessel density and detection of angiogenic hotspots in histological whole-slide images

**DOI:** 10.18632/oncotarget.4383

**Published:** 2015-06-08

**Authors:** Jakob Nikolas Kather, Alexander Marx, Constantino Carlos Reyes-Aldasoro, Lothar R. Schad, Frank Gerrit Zöllner, Cleo-Aron Weis

**Affiliations:** ^1^ Institute of Pathology, University Medical Centre Mannheim, University of Heidelberg, Mannheim, Germany; ^2^ Computer Assisted Clinical Medicine, Medical Faculty Mannheim, University of Heidelberg, Mannheim, Germany; ^3^ School of Engineering and Mathematical Sciences, City University London EC1V OHB, United Kingdom

**Keywords:** tumor angiogenesis, digital pathology, spatial statistics, vessel density

## Abstract

Blood vessels in solid tumors are not randomly distributed, but are clustered in angiogenic hotspots. Tumor microvessel density (MVD) within these hotspots correlates with patient survival and is widely used both in diagnostic routine and in clinical trials. Still, these hotspots are usually subjectively defined. There is no unbiased, continuous and explicit representation of tumor vessel distribution in histological whole slide images. This shortcoming distorts angiogenesis measurements and may account for ambiguous results in the literature.

In the present study, we describe and evaluate a new method that eliminates this bias and makes angiogenesis quantification more objective and more efficient. Our approach involves automatic slide scanning, automatic image analysis and spatial statistical analysis. By comparing a continuous MVD function of the actual sample to random point patterns, we introduce an objective criterion for hotspot detection: An angiogenic hotspot is defined as a clustering of blood vessels that is very unlikely to occur randomly. We evaluate the proposed method in *N*=11 images of human colorectal carcinoma samples and compare the results to a blinded human observer. For the first time, we demonstrate the existence of statistically significant hotspots in tumor images and provide a tool to accurately detect these hotspots.

## INTRODUCTION

Tumor angiogenesis is the growth of blood vessels from healthy tissue into tumor tissue [1]. Virtually all solid tumors in humans require angiogenesis for growth beyond a minimal size [2]. One way to observe angiogenesis is through histological tumor sections, where blood vessel profiles can be assessed by immunostaining for endothelial markers like CD31 or CD34 [3]. Microvessel density (MVD) and vessel distribution have been used to describe and compare the vascularization of different tumors. For example, vascularization patterns differ in breast carcinomas and sarcomas [4]. Similarly, renal cell carcinomas and breast carcinomas differ in terms of MVD [5]. The different vascular patterns in different tumors have led to biologically, and potentially clinically, relevant conclusions. Yet, most of these findings are based on vessel counting in subjectively defined regions and have not been validated by objective whole-slide image analysis methods [6, 7].

Generally, tumor microvessel density (MVD) is seen as an independent prognostic factor in several cancer entities. However, there are still conflicting results with respect to MVD as an independent prognostic factor. For example in breast cancer, several studies in the last 20 years have confirmed the original study from 1992 that found a correlation of MVD to prognosis [8-10]. In contrast, a recent reevaluation could not reproduce these results [11]. For colorectal carcinoma, a large number of studies investigated the prognostic relevance of intratumoral MVD: Although a meta-analysis showed that MVD is indeed a prognostic factor for relapse-free and overall survival, the individual study results vary considerably [12]. In prostate cancer, the role of MVD as an independent prognostic factor is unclear: In a large study, manually counted MVD was correlated to tumor aggressiveness and was a predictor of prostate-specific antigen (PSA) recurrence after initial prostatectomy, but was not an independent prognostic factor [13]. However, another large study could identify prostate cancer MVD as an independent prognostic factor in subjectively defined representative hotspot areas [14]. Yet another study reported that MVD in prostate cancer compared to MVD in normal prostate tissue is not elevated at all [15]. Taken together, there are contradictory results on the importance of MVD in breast cancer, colorectal cancer and prostate cancer.

A reason for these ambiguities could be a methodological shortcoming of most approaches for MVD quantification. All common approaches are based on two steps: First, a highly vascularized region within the tumor is identified subjectively according to Weidner's criteria [16]. This region is considered the “angiogenic hotspot”. Second, tumor vessels are counted only in this region, either by random sampling or by counting all vessels within this region [6]. One of the most widely used methods for MVD quantification is Chalkley counting [17]. This method has been shown to be fairly reproducible but still relies on prior subjective identification of a hotspot area [18].

Correspondingly, the most recent international consensus paper on angiogenesis quantification (from 2002) states: “[...] reliable angiogenesis parameters are urgently needed in the emerging therapeutic setting of anti-angiogenesis.” [19]. Following this conclusion, some authors have used state-of-the-art image analysis methods to quantitatively analyze the tumor vasculature. For example, Mikalsen and co-workers have used automatic image analysis and linked tumor vessel morphology to breast cancer patient outcome [20]. Yet, the authors of that study also selected angiogenic hotspot areas manually.

Interestingly, for some tumor entities it is still debated whether hotspots actually exist. For example for sarcoma, one study quantified MVD in subjectively selected hotspot areas [21] while another study stated that vessels in sarcoma samples are homogenously distributed and do not show any clustering [4].

In summary, there is a need to replace manual hotspot selection in MVD analysis of tumor samples. Also, the concept of tumor angiogenic hotspots has to be questioned more fundamentally. To the best of our knowledge, this concept has never been challenged since Weidner et al. have proposed it in 1991 [16]. Until today, the concept of angiogenic hotspots is only grounded on subjective observation of tumor vessel distribution in a histological slide. The only study known to us that investigated automatic vascular hotspot selection is more than 15 years old. In this study, blood vessels counts were aggregated in cells of a coarse grid and no spatial statistics were used to verify possible clustering [22].

The purpose of the present study is to close that conceptual gap in quantitative tumor vessel analysis. For the first time, we formally address the question whether tumor angiogenic hotspots are merely local fluctuations in a random pattern or occur in a statistically significant manner. Furthermore, we propose an explicit definition of tumor angiogenic hotspots based on spatial statistical models. To achieve this, we take three steps: i) We elaborate a concept of continuous mapping instead of gridded counting of blood vessels in histological whole slide images; ii) We present a novel explicit model for vessel clustering and provide an objective optimal criterion of tumor angiogenic hotspots and iii) We use spatial statistical models to compare tumor vessel distribution to random point patterns.

## RESULTS

### Tumor vessel density can be represented by a continuous density function

**Figure 1:**
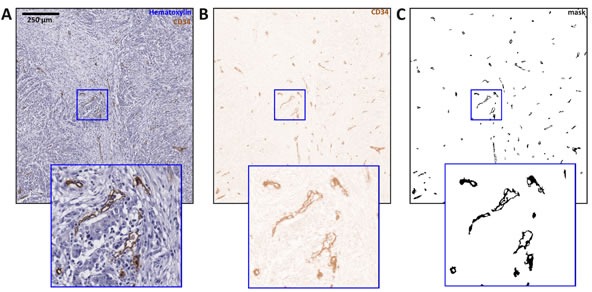
Immunostained blood vessels are automatically segmented in whole-slide images In this figure, image tiles of 1600×1600 px are shown and an image detail is enlarged. **A.** Original image region, **B.** result of color deconvolution, **C.** result of thresholding and morphological post-processing.

**Figure 2:**
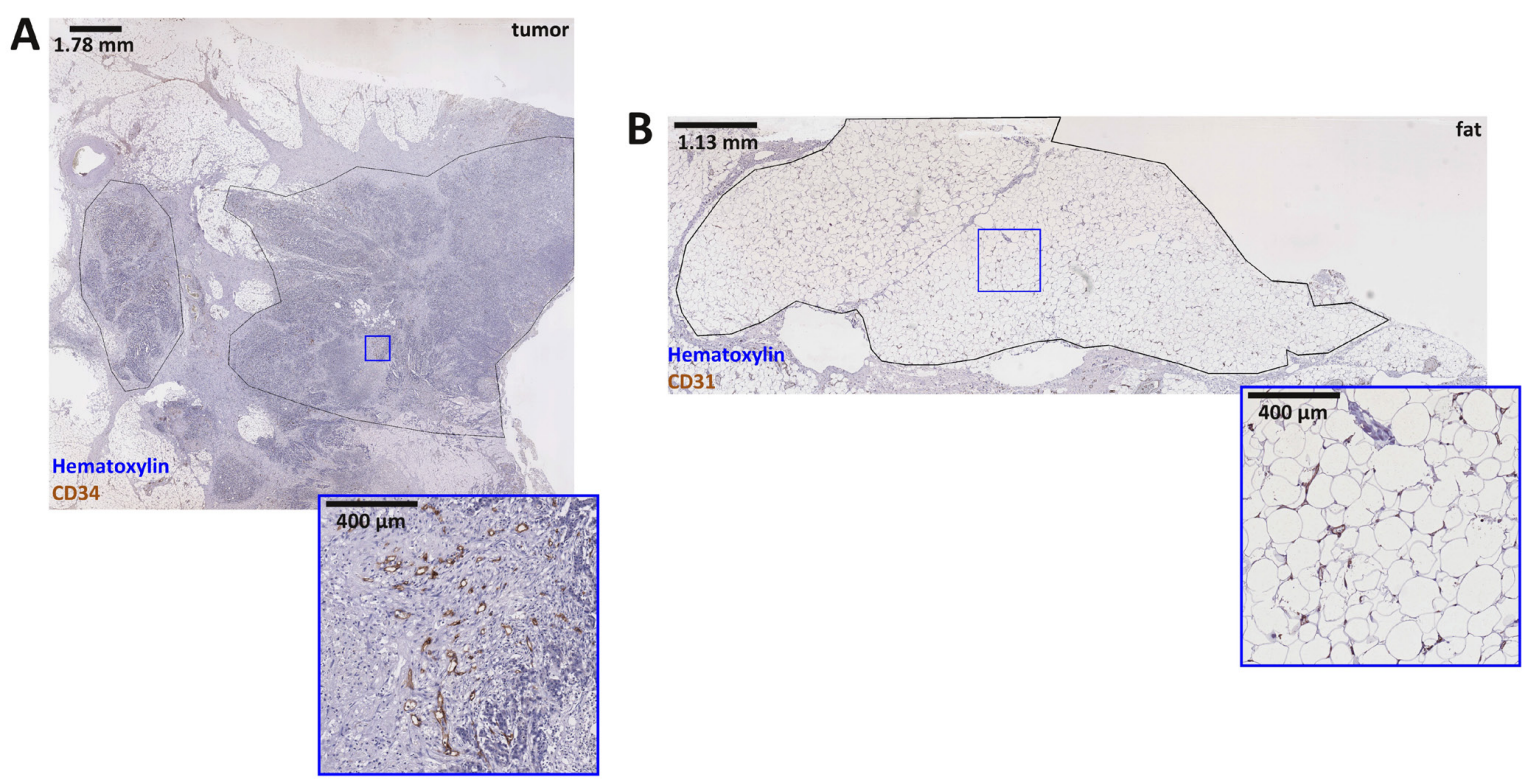
Whole slide image of a colorectal tumor sample Panel **A.** shows a whole slide image of a colorectal cancer sample, two tumor regions are delineated by hand. Analysis of the right-hand region is subsequently shown in Figure. 4. Below, a detail is enlarged to demonstrate the staining quality. In panel **B.** a fat tissue sample is shown. The delineated fat tissue region served as a negative control for validation of the new method. Below the main panel, a detail is enlarged. The corresponding blood vessel map can be found in Figure 3.

**Figure 3:**
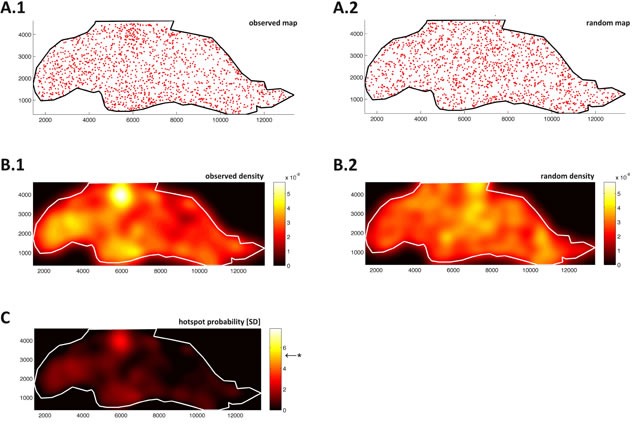
Blood vessels in fat tissue are distributed randomly and do not show significant clustering A point map of vessels in fat tissue from Figure 2B is shown in panel **A.1.** Point map of random pattern **A.2.** in the same region; microvessel density function of fat tissue **B.1.**; density function of the complete spatial randomness (CSR) model **B.2.**; color-coded units of the density functions are arbitrary. **C.** Probability map (units: standard deviations of CSR), Bonferroni corrected level of significance is at *F* = 5.25 (marked by *). No significant angiogenic hotspot can be detected in this tissue region.

**Figure 4:**
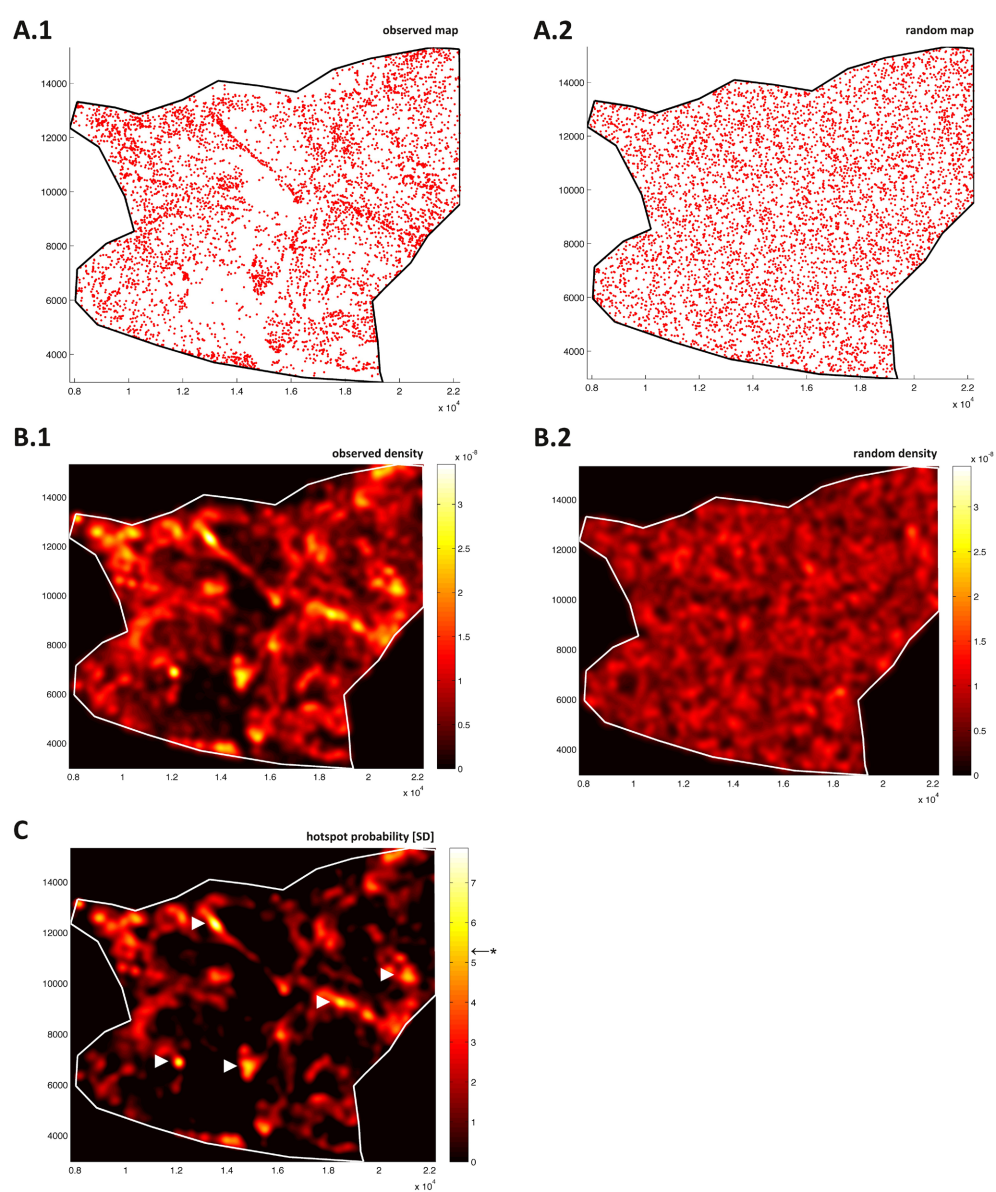
Blood vessels in colorectal tumor tissue show highly significant clustering A point map of vessels in colorectal tumor tissue from Figure 2A is shown in **A.1.** Point map of random pattern **A.2.** in the same region; microvessel density function of tumor tissue **B.1.**; density function of the complete spatial randomness (CSR) model **B.2.**; color-coded units of the density functions are arbitrary. **C.** Probability map (units: standard deviations of CSR), Bonferroni corrected level of significance is at *F* = 5.27 (marked by *). Five statistically significant tumor angiogenic hotspots emerge in this region (indicated by arrows).

Conventionally, angiogenic hotspots are manually selected in histological tumor samples. This manual procedure is inherently biased and inaccurate. By using a new automatic method, *N* = 11 tumor tissue images from *N* = 9 patients were analyzed for the presence of angiogenic hotspots. First, CD34-immunostained whole slides of colon carcinoma samples were scanned and blood vessels were extracted by image segmentation (Figure 1). Tumor tissue in the whole slide image was manually delineated (Figure 2A) and blood vessels within this region of interest (ROI) Ω_i_ were extracted. This was also done for fat tissue, which was used as a negative control for validation of the procedure (Figure 2B). We did not use normal colon mucosa as a control because it consists of multiple non-solid glands and therefore would yield an intrinsically non-random and anisotropic vascularization pattern. For a given map subset in each ROI Ω_i_, a corresponding random point pattern was created according to a complete spatial randomness (CSR) process (Fat: observed pattern in Figure 3A.1, random pattern in Figure 3A.2; Tumor: observed pattern in Figure 4A.1, random pattern in Figure 4A.2). This corresponding CSR pattern constituted an intrinsic control for each tissue sample because it had the same ROI geometry and overall particle density as the observed pattern. By using kernel density estimation (KDE), density functions were calculated for these point patterns: function $\rho_{\mathrm{obs}}(x,y)$ for the observed pattern and function $\rho_{\mathrm{CSR}}(x,y)$ for the CSR pattern (Fat: Figure 3B.1 and Figure 3B.2, Tumor, Figure 4B.1 and Figure 4B.2). To the best of our knowledge, these spatial statistical methods have never been applied in the context of tumor vessel density before.

### A hotspot probability map shows non-random fluctuations of tumor vessel density

Observing the point patterns and corresponding density functions, it can be seen that density fluctuations are present both in $\rho_{\mathrm{obs}}$ and $\rho_{\mathrm{CSR}}$. While in fat tissue, these fluctuations were of the same magnitude in the observed pattern (Figure 3B.1) and CSR pattern (Figure 3B.2), this was not the case for the tumor tissue: In tumor tissue, the density fluctuations are much more pronounced in $\rho_{\mathrm{obs}}$ (Figure 4B.1) than in $\rho_{\mathrm{CSR}}$ (Figure 4B.2). This suggested that non-random hotspots existed in tumor tissue but not in fat tissue. To quantify this non-randomness, a probability function F(x,y) was defined by normalizing $\rho_{\mathrm{obs}}$ to $\rho_{\mathrm{CSR}}$. The density of the CSR distributed pattern was considered noise and therefore its mean function value $\mu_{\mathrm{CSR}}$ was subtracted from the observed density function $\rho_{\mathrm{obs}}(x,y)$. By normalizing the resulting function to the standard deviation $\sigma_{\mathrm{CSR}}$ of $\rho_{\mathrm{CSR}}(x,y)$, a continuous hotspot probability function

[1] $$F(x,y)=\frac{\rho_{\mathrm{obs}}(x,y)-\mu_{\mathrm{CSR}}}{\sigma_{\mathrm{CSR}}}$$

was defined on the same grid as $\rho_{\mathrm{obs}}(x,y)$. Thus, the function value F(x,y) at each location within Ω_i_ was a measure of the degree by which the microvessel density at this particular location deviated from the density expected under the null hypothesis of CSR. The degree of non-randomness was expressed in multiples of standard deviations of a random point pattern in Ω_i_. According to the inverse standard normal cumulative density function (CDF)

[2] $$\rho (p)=\frac{1}{\sqrt{2\pi }}\int_{-\infty }^{p}e^{-\frac{t^{2}}{2}}dt$$

all values of $F(x_{h},y_{h})\geq 1.6$ were unlikely to occur under the null hypothesis (with a *p*-value of 0.05). Consequently, the point $x_{h},y_{h}$ was considered to be part of a significant hotspot. However, because $\rho_{\mathrm{obs}}(x,y)$ and therefore F(x,y) were sampled at a large number of sampling points (approx.10^5^ to 10^6^), a correction for multiple testing had to be applied. We chose Bonferroni correction because this method yields a conservative estimate, which makes false positive results extremely unlikely. Combining the inverse standard normal CDF with Bonferroni correction at n sampling points, the threshold above which a point $x_{h},y_{h}$ was considered to be part of a significant hotspot (with a *p*-value of 0.05/n) increased to

[3] $$F(x_{h},y_{h})\geq \frac{1}{\sqrt{2\pi }}\int_{-\infty }^{\frac{0.05}{n}}e^{-\frac{t^{2}}{2}}dt$$

This hotspot probability map was computed for a sample fat tissue region (Figure 3C), where no significant hotspots emerged and for a sample colon tumor region (Figure 4C), where five contiguous non-random hotspot regions emerged.

### Angiogenic hotspots are detected in each item of a series of colon tumor tissue samples

After calculating the hotspot probability map for a sample colon tumor image and a sample fat tissue image, we analyzed a series of *N* = 10 colon tumor samples from *N* = 8 patients (Table 1). Again, automatically created corresponding CSR patterns served as an intrinsic control for each sample. We found that within the angiogenic hotspot areas, average MVD was 520 vessels per mm^2^, while in the whole tumors, average MVD was 89 vessels per mm^2^ (Table 1). The number of hotspots per sample varied largely, indicating a high biological variation between the samples. Still, all tumor images yielded statistically significant angiogenic hotspots and were significantly different from corresponding CSR patterns. The absolute values for MVD were compatible with prior studies. For example, Bossi et al. reported a (manually counted) MVD of 115 ± 39 vessels per visual field (181 ± 61 vessels per mm^2^) in highly vascularized areas in 178 colorectal carcinomas [23]. This count is higher than the average MVD for whole tumors and lower than hotspot MVD found in the present study. This discrepancy is probably due to the fact that hotspots in Bossi's study were per definition the size of a visual field (0.64 mm^2^) while hotspots in the present study were not restricted to a fixed size and thus more accurately traced the actual hotspot boundaries. Average hotspot size found in the present study was on average 0.08 mm^2^ (see, for example, hotspots in Figure 4C compared to the dimensions in Figure 2A).

**Table 1:** Measurements for all analyzed colon tumor samples In this table, various automatic measurements are listed for all 11 analyzed colon tumor whole slide images. A blinded observer delineated primary and secondary hotspots in image 1 to 10. Image 0 was not evaluated because the observer had seen it before. In the last two columns, the proportion of these hotspots also detected by automatic analysis is listed. Abbreviations: HS = hotspot, MVD = microvascular density, SD = standard deviation, KDE = kernel density estimation, xbar = column mean, n.d. = not determined

tumor image	tumor size	HS count	mean MVD in tumor	mean MVD in HS	SD MVD in HS	KDE bandwidth	hotspot density	primary HS overlap	secondary HS overlap
*ID*	*mm^2^*	*count*	*vessels per mm^2^*	*vessels per mm^2^*	*vessels per mm^2^*	*px*	*hotspots per mm^2^*		
**0**	85	5	66	446	228	157	0.1	n.d.	n.d.
**1**	116	11	101	544	142	128	0.1	1 of 1	1 of 1
**2**	132	35	125	641	325	105	0.3	1 of 1	4 of 4
**3**	59	28	122	809	433	85	0.5	1 of 1	1 of 2
**4**	68	28	67	438	214	94	0.4	1 of 1	0 of 2
**5**	116	42	48	318	255	121	0.4	1 of 1	3 of 3
**6**	74	46	82	478	210	95	6.0	1 of 1	2 of 2
**7**	90	25	145	756	257	99	0.3	1 of 1	0 of 0
**8**	48	13	51	374	333	144	0.3	0 of 1	1 of 2
**9**	200	127	81	435	198	90	6.0	1 of 1	1 of 1
**10**	62	7	89	475	180	141	0.1	1 of 1	0 of 1
			**xbar = 89**	**xbar = 520**		**xbar = 114**		**9 of 10**	**13 of 18**

### The novel spatial statistical approach is valid compared to established statistical models

We propose a new method for detecting non-random fluctuations in two-dimensional point patterns. To the best of our knowledge, such a method has never been used before, even outside the realm of digital pathology. Therefore, we validated the results using several spatial statistical methods implemented in the well-established R package Spatstat [24]. For a given point pattern, the empty space *F*(*r*) function *G*(*r*) and nearest neighbor distance distribution function were calculated and compared to the null hypothesis of complete spatial randomness (CSR). In accordance with our results, these well-established statistical methods did not yield any non-random clustering for fat tissue (Figure 6A) while non-random clustering was detected in tumor tissue (Figure 6B). These findings suggest that the new spatial analysis method is a valid approach to detect non-random fluctuations in point patterns. However, it does not necessarily follow that the new method validly detects angiogenic hotspots in whole slide images of tumors. Therefore, as will be explained below, we used a different approach to validate the hotspot detection by the proposed new method.

### Automatic hotspot detection largely coincides with manual hotspot detection by a blinded observer

**Figure 5:**
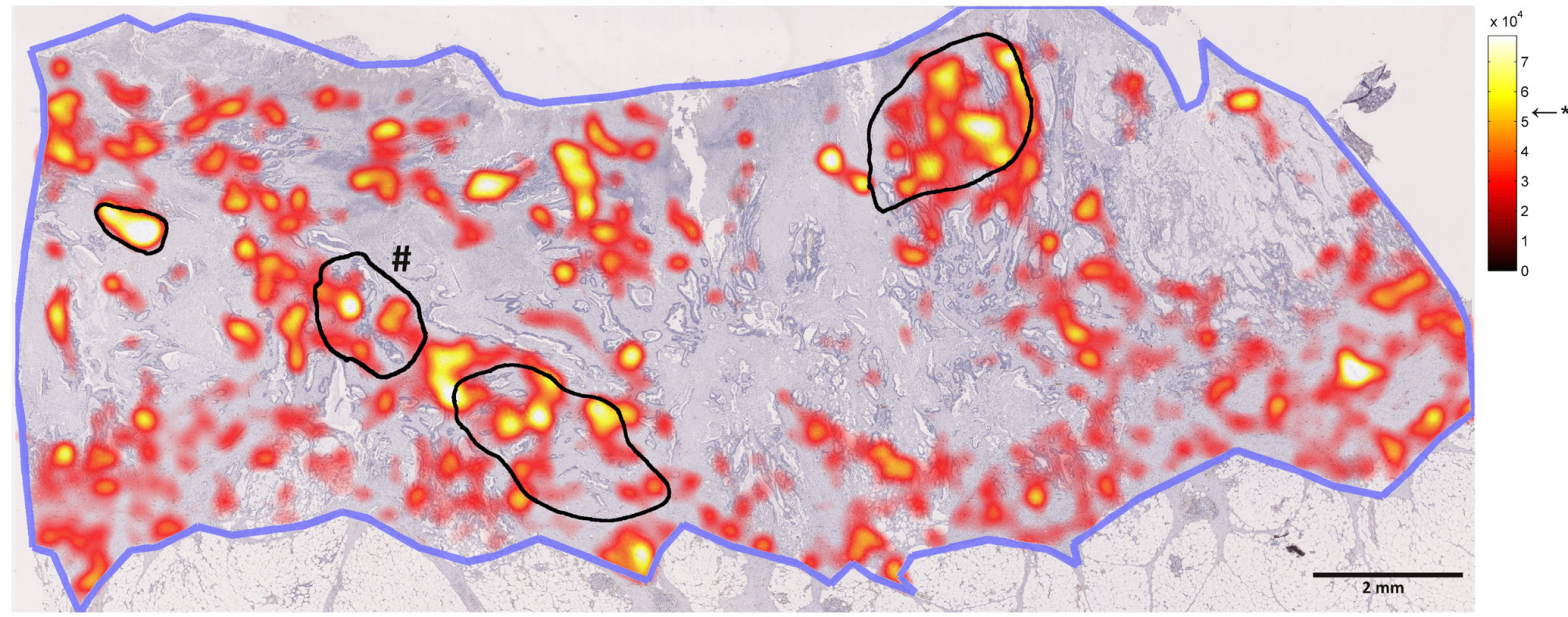
Comparison of manual and automatic hotspot detection In this figure, a whole slide image is shown in low magnification. The blue line shows the contour of the tumor. The black lines show angiogenic hotspots as delineated by a blinded human observer (# marks the primary hotspot as defined by the observer). The hotspot probability map is overlaid (red/yellow; level of significance is indicated by *). It can be seen that all manually detected hotspot areas were also detected by the automatic method.

Generally, whenever a new method is introduced, it should be compared to an existing gold standard. However, for angiogenic hotspot detection, the present gold standard is subjective evaluation by a pathologist and our method is conceptually different. Conventionally, pathologists subjectively identify exactly one angiogenic hotspot in a given slide according to Weidner's method [16]. Our proposed method does not reproduce this selection of exactly one angiogenic hotspot. Instead, each point in the whole slide image is assigned a probability value of being part of a significant vessel cluster. Thus, one, several or none of these significant clusters can be present in a given image. We therefore chose to validate the proposed method as follows: a blinded observer (CAW) manually delineated a primary angiogenic hotspot in all images according to Weidner's method [16]. Optionally, the observer delineated secondary angiogenic hotspots. We then compared these regions to the automatically detected hotspot areas and checked whether these regions overlapped (any degree of overlap). We found that subjectively detected primary hotspots overlapped automatically detected hotspots in 9 of 10 cases while subjectively detected secondary hotspots overlapped automatically detected hotspots in 13 of 18 cases (Table 1, Figure 5).

**Figure 6:**
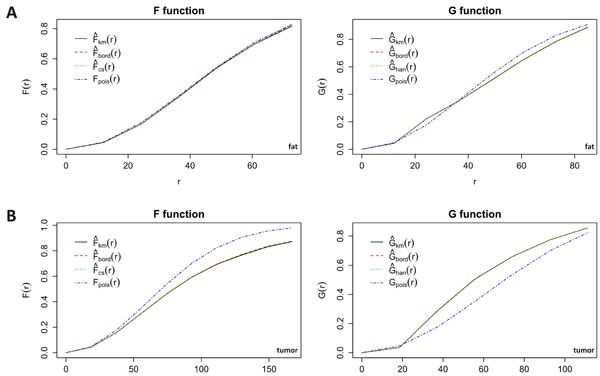
Spatial statistics for fat and tumor Empty space function *F*(*r*) and nearest neighbour distance distribution function *G*(*r*) for vessels in fat **A.** and tumor **B.** tissue. The observed functions are plotted against the functions of a corresponding random pattern. While F and G for fat do not differ from the random functions, tumor vessel distribution markedly differs from a random pattern. km = Kaplan-Meier estimate, cs = Chiu-Stoyan estimate, bord = border corrected estimate, han = Hanisch estimate, pois = theoretical Poisson distribution (CSR).

#### Synopsis of major findings

In the present study, we present the first continuous hotspot probability map for evaluation of histological whole slide images. Our method explicitly assigns a probability value to each point on the source image. This value gives the probability by which an angiogenic hotspot is present at the respective location in the source image. Thus, our method yields three main results: First, it tells us whether blood vessels in a given tissue sample are randomly distributed or form statistically significant hotspots. Second, these hotspots can be accurately localized within the image. Third, although the decision if a given point is part of a hotspot is a binary decision, each point is assigned an exact probability value. Thus, the level of significance can be adjusted to change the extent of an angiogenic hotspot in a given setting. In the present manuscript, we chose a very conservative approach for hotspot detection, effectively ruling out false positive results. In different applications, it might be appropriate to lower the level of significance for hotspot detection, thereby increasing the number and the size of detected hotspots at the cost of potential false positive results.

## DISCUSSION

### The concept of tumor angiogenic hotspots revisited

The notion of vessel clustering in angiogenic hotspots in histological samples of solid tumors is widely taken for granted, but to the best of our knowledge, it has never been challenged by means of spatial statistics. The alternative hypothesis that angiogenic hotspots are a result of local fluctuations in random blood vessel distribution has not been tested yet. Furthermore, for angiogenesis researchers and pathologists, a naturally derived definition of an angiogenic hotspot and a tool for hotspot probability mapping has not been available so far.

In the present study, we describe such a tool and, based on it, show for the first time that blood vessels in colorectal carcinoma are not randomly distributed but show statistically significant clustering representing angiogenic hotspots. Our findings contribute to the concept of angiogenic hotspots in three ways: i) by a new notion of continuous quantitative analysis of histological imaging independent of data aggregation in grid cells; ii) by the explicit definition of tumor angiogenic hotspots in histological whole slide images and iii) by the verification of significant vessel clustering by using spatial statistical models.

### Comparison to previous approaches for vessel density mapping

One achievement of the method presented in this study was automatic generation of vessel maps for whole-slide tumor images. Several groups have presented similar methods in the past. For example, vessel segmentation was performed on whole slide images of experimental tumors in a study from 2003 [25]. In two studies, vascular density was visualized in whole-slide images of prostate cancer sections [26, 27]. Another study reported the use of whole-slide analysis of immunofluorescence positivity for vascular markers without morphologically analyzing individual vessel profiles [28]. Peritumoral density of lymphatic vessels was automatically analyzed by Balsat *et al*. [29]. A recent approach included segmentation of lymphatic vessels by proprietary software and analysis of global measures of vascularization [30]. In a different study, proprietary software was used to detect immune cell infiltrates in colorectal carcinoma samples and to map these infiltrates to whole-slide images [31].

Although the automatic generation of vessel maps is not new, all subsequent steps of the present study are unique in the context of MVD assessment: To analyze MVD, we use a continuous density estimation approach. Previous studies aggregated counts on a grid, making the results dependent on grid translation or rotation. Furthermore, the method described in the present study uses an intrinsic control pattern for each sample, so that vascularization patterns are pairwisely compared to an appropriate null hypothesis. This solves the problem of choosing a suitable control tissue for a tissue of interest.

In the next section, we will compare our approach to previously described methods in more detail. First, we will consider previous approaches to evaluate MVD in whole slide images; then, we will consider previous approaches to elucidate the concept of angiogenic hotspots.

### Comparison to previous approaches for tumor angiogenic hotspot detection

The classical definition of a tumor angiogenic hotspot has been provided by Weidner and co-workers, who defined a hotspot as the subjectively chosen “area of highest neovascularization” within a tumor [16]. Still in recent studies, Weidner's definition is used as a basis for MVD quantification [32]. By this definition, there is exactly one hotspot area in any sample, although significant vessel clustering can be present in more than one region. This may yield false negative results.. Conversely, this method also yields false positive results because hotspot areas are found in samples without any statistically significant clustering. In the present study, we present the first objective definition of tumor angiogenic hotspots. Our definition is not based on subjective assumptions but on spatial statistical models. If there is no statistically significant blood vessel clustering in a given tissue sample, our method does not identify any hotspot areas. Conversely, if more than one contiguous region shows significant vessel clustering, our method identifies these regions. By defining a continuous hotspot probability map for a whole slide tumor image, the statistical significance of hotspot areas can be quantitatively compared within a given sample and also between samples. Thus, for the first time, it becomes possible to quantitatively compare the presence, number and extent of angiogenic hotspots between different tissue samples.

### Implications for theoretical models of tumor metabolism and structure

It is known that molecularly and morphologically, tumor tissue is highly heterogeneous [33-35]. Tumor cell proliferation, tumor cell metabolism and tumor angiogenesis are subject to pronounced heterogeneity within a solid tumor [36, 37]. This heterogeneity is generally not considered in studies quantifying MVD. Therefore, the findings presented in this study are relevant for theoretical models of tumor heterogeneity. Most models of cell metabolism, immune response and drug distribution are dependent on the pattern of tumor vascularization [38]. How exactly this tumor vascularization pattern varies locally has never been explicitly investigated. In tumor angiogenesis research, it is assumed that tumor vascularization is generally higher at the tumor margin when compared to the hypoxic tumor center (e.g. for colorectal carcinoma [39]); but this assumption is based on subjective and/or random sampling of regions of interest and has not been validated by use of spatial statistics and independent of a coarse grid. Quite strikingly, analysis of *N* = 11 tumor images performed in the present study showed that angiogenic hotspots can be found close to the tumor margin but are also located close to hypovascularized regions in the tumor center (see, for example, Figure 4C).

Thus, the angiogenic hotspot probability map which we present in this study could form the basis for refinement of existing models of solid tumors [37]. For example, drug distribution measurements in experimentally induced tumors in mice could be matched to angiogenic hotspot maps of the same tumors to generate more realistic assumptions for models of drug distribution. Also, as implied in Suppl. Figure 1, tumor cell proliferation visualized by Ki67 could be quantitatively assessed in whole slide images and be matched to angiogenic hotspot probability maps to generate new insights for more accurate theoretical models of solid tumors.

### A novel approach for quantitative analysis of histological slides: implications for histopathology

Objective, quantitative image analysis in histology is a key prerequisite for individualized cancer therapy [40]. Assessment of tumor MVD is performed using different methods like manual counting or Chalkley count that are not objective and cannot be used in a high-throughput manner [6]. In the present study, we define a continuous hotspot probability function in histological slides, which is a fundamentally new notion of quantitative analysis of histological slides. In histopathological routine, quantification of MVD is often performed either in randomly sampled high power fields (HPFs) or in subjectively selected HPFs within areas of interest, for example angiogenic hotspots [19]. A more objective approach used in quantitative analysis is to divide the specimen into a regular grid and count blood vessels in each cell of this grid. This has been done manually [22] and automatically [27]. While these grid-based methods are useful for exploratory analysis of tumor MVD, they are not suitable to automatically detect hotspots. Tumor MVD distribution defined on a regular grid is not invariant with respect to grid cell size and grid translation or rotation. The algorithm we use in the present study is different: For computational reasons, whole slide images are divided into tiles for image segmentation, but a tile overlap ensures that the segmentation result is invariant with respect to tile dimensions. We then define a continuous hotspot probability map which is computationally represented at approx. 10^6^ sampling points and does not require arbitrary grid cell parameters. The single most important parameter in our model is kernel bandwidth used to determine the density function and this parameter is optimally estimated for each sample according to a well-established algorithm [41].

The paradigm shift we propose for quantification of MVD also bears consequences for other areas of quantitative histopathology. For example, random sampling or subjective placement of HPFs is used to measure parameters like the Ki67 index in histological tumor samples. These quantitative measurements have profound consequences for therapy and prognosis. Still, these measurements methods are partly error-prone and there is no rigorous definition and no scalable tool for their detection in a more or less heterogeneous tumor sample. The method for continuous probability mapping of angiogenic hotspots presented in this study could be a starting point to establish similar methods for other target variables in routine histopathology, thereby extending beyond MVD quantification.

To implement our findings in histopathological routine, we would envision the incorporation of the presented method in digital pathology workflows. For example, a digital pathology workstation could enable the pathologists to select a tumor tissue of interest in a given whole slide image. Then, angiogenic hotspots could be automatically delineated (including a *p*-value for each hotspot) and MVD within these hotspots could be recorded. Another possibility would be double immunostaining for endothelial markers and proliferation markers, so that proliferating angiogenesis could be selectively monitored.

### Integrating global and local aspects of spatial analysis

While in histopathology, spatial statistical methods are not widely used, these methods are well established in other fields such as geography or epidemiology. Conventionally, global measures for particle clustering are obtained by methods to detect whether significant clustering or dispersion is present in a pattern, for example by measuring distances between particles or by measures of autocorrelation. While many classical methods rely on aggregation of spatial variables into grid cells, k-means clustering and other methods do not aggregate data before analysis. For example, the latter was used in image analysis of histological slides to detect Ki67 hotspots [42]. The novel method we present in this study is different to previously presented methods: The whole pattern is taken into account and compared against a null hypothesis of a homogenous two-dimensional Poisson process. Then, the density is assessed locally and by normalizing to the null hypothesis, a probability map for the presence of non-random clustering is generated (Figure 3C for fat, 4C for tumor). Thus, without using binning or other means of aggregation, it can be determined whether statistically significant clustering is present in the dataset and where these clusters are located.

### Limitations and perspectives

Although the tumor vasculature extends in three-dimensions, vessel distribution is commonly assessed in two-dimensional slices. Thus, objects in histological images are randomly or systematically sampled from a larger population. This 2D sampling may introduce errors into quantitative methods in histopathology, including the method presented in the present study. To reconstruct a 3D pattern from observations in a plane, serial section reconstruction is one of the most widely used techniques [43]. It would therefore be possible to measure vessel distributions in serial, registered sections of tumor samples and derive a three-dimensional hotspot probability map. However, in the present study, we restrict ourselves to the 2D case since serial sections and subsequent 3D-reconstruction are not yet relevant for clinical histopathology. Furthermore, the methodological implications arising from a third dimension are not negligible (e.g. vessel segmentation and statistical comparison of distributions in three dimensional space).

In order to automatically create vessel hotspot probability maps in clinical histopathology, two further possible issues would have to be controlled: First, MATLAB implementation of the presented algorithm is well suited for prototype building but not readily applicable in routine histopathology. Second, we performed a manual quality check of the stained slides and requested new stainings for macroscopically folded or torn tissue samples. For a fully digital pathology workflow, this quality check should be done by automatic image analysis methods.

Still, our study opens up another intriguing perspective: By turning from microscopic structures like single, small vessels to angiogenic hotspots of a certain size and distribution, it seems to be possible to change the measurement scale from μm to mm. Consequently, histological vascular patterns could be compared with radiological data (e.g. tumor perfusion). Also, it would be possible to combine our approach with new techniques using high resolution Magnetic Resonance Microscopy [44].

### Summary

In this study we present a new method to create continuous angiogenic hotspot probability maps of histological whole slide images automatically and with reasonable computational efficiency. We believe that this tool will prove useful to further develop and challenge theoretical models of tumor biology, especially in the field of tumor angiogenesis research. Furthermore, the presented approach may advance digital quantitative histopathology with regard to personalized cancer diagnosis.

## MATERIALS AND METHODS

### Tissue sample acquisition, staining and digitization

Tissue specimens of formalin-fixed paraffin-embedded human tumors were retrieved from the pathology archive. We retrieved *N* = 9 colon carcinomas (each from a different patient). Seven tumors yielded one tissue slide, one tumor yielded two tissue slides and one tumor yielded an unusually large tissue slide that had to be split in two parts. All resulting slides were completely separated and did not overlap in any dimension. Therefore, in total, we analyzed *N* = 11 independent colorectal cancer images (Table 1). A pathologist (CAW) confirmed the diagnosis independently of the original report and checked the tissues slides for artifacts such as torn tissue or unspecific staining artifacts. Also, a sample of fat tissue was used for initial validation of the procedure, as described above. The specimens were CD31, CD34 and Ki67 immunostained (DAB) with hematoxylin background staining using a routine immunoperoxidase technique (CD31: DakoCytomation M0823, 1:500; CD34: Immunotech PNIMO786, 1:500; Ki67: DakoCytomation M7240, 1: 800; pH 6, 40 minutes). We found that CD31 staining of colon carcinoma tissue labeled high numbers of CD31-expressing immune cells (e.g. macrophages) in addition to endothelial cells in the tumor tissue ROIs. Therefore, for colorectal carcinoma samples, we used CD34 staining that did not label non-endothelial structures in the carcinoma regions of the tissue samples we studied. Subsequently, the slides were fully digitalized using an Aperio ScanScope (Aperio/Leica biosystems) and saved as compressed Aperio svs files, typically yielding 300 MB per slide. All experiments were in accordance with the Declaration of Helsinki and all image processing methods were in accordance with Digital Image Ethics [45]. All images were anonymous and use of patient samples complied with the guidelines of the institutional reviewer board.

### Whole-slide image segmentation and post-processing

The basis for this spatial analysis of vascularization pattern in histological whole-slide images is to segment blood vessels in a fully automatic, unbiased and reliable fashion. To achieve this, we combined various well-described and mature image-analysis techniques. A core component is the morphological post-processing algorithm published by Reyes-Aldasoro et al. [46, 47]. In the present study, we expanded this algorithm to whole slide images by a tesselating the large image into overlapping tiles and by subsequently rearranging the whole slide image. Thus, the computationally expensive procedure of object recognition was successively applied to sub-images and could be run on a desktop workstation (see also Suppl. Figure 2). The following image-segmentation steps were performed: a) A whole slide image was tessellated into 1600 × 1600 pixels sections, each of which was processed separately (Figure 1A). b) Ruifrok's color deconvolution [48] was applied to extract the brown (DAB / CD31 or CD34) channel (Figure 1B). c) The brown channel was thresholded using Yen's automatic threshold detection method [49]. d) Morphological post-processing steps as described by Reyes-Aldasoro [46, 47] were applied to join nearby objects, split objects and close gaps within objects (Figure 1C). e) Objects smaller than a minimal size (default: 65 pixels) were discarded from the analysis as these objects were considered as noise (e.g. single CD31-positive non-endothelial cells, for example macrophages). A flowchart of the computational algorithm is shown in Suppl. Figure 2.

### Computational implementation of image segmentation

To scale and divide whole slide images, we used the open-source tool VIPS/Nip2 version 7.40.2 (Imperial College London, UK), which is well suited to handle extremely large images > 100 MB [50]. For color deconvolution and Yen segmentation, we wrote a macro for Fiji/ImageJ version 1.48s with Java version 1.6.0 (http://fiji.sc/About) making use of Fiji plugins [51]. MATLAB programming was used for all subsequent data analysis steps (MATLAB R2014b, Mathworks, Natick, MA, USA). For technical details on computational analyses, see supplements (“Details of the computational procedures” and Suppl. Figure 2).

### Spatial analysis of tumor blood vessels

After all blood vessels in a histological whole slide image were identified, their centroid coordinates were saved for further processing. Approximately 10,000 to 50,000 blood vessels were detected per whole slide image, yielding a dataset of approximately 5 MB. This highly condensed representation of blood vessels in a histological image made it possible to perform spatial statistical analysis of whole slide datasets. Since a whole slide image typically contains several tissue types (tumor and non-tumor), a pathologist (CAW) manually delineated one or more polygonal regions of interest (ROIs) in each whole-slide image (Figure 2A and 2B). ROIs did not contain any torn tissue regions or staining artifacts. ROI coordinates were transferred to the vessel map and all vessels within the ROI were saved as a map subset.

### Comparison of observed patterns to the null hypothesis

Blood vessel distribution in each map subset was compared to the null hypothesis of complete spatial randomness (CSR). To achieve this, a random point pattern was created in each ROI by a homogenous two-dimensional Poisson process. This pattern contained the same number of objects as the observed pattern so that the overall density within each ROI did not vary between the observed pattern and the random pattern. This random point map was used as an intrinsic internal control for each observed vessel map and vessel density of the observed pattern was normalized to vessel density of the random pattern.

### Kernel density estimation and probability mapping

KDE included convolution of the dataset with a Gaussian kernel, yielding a density function $\rho_{\mathrm{obs}}(x,y)$. This density function $\rho_{\mathrm{obs}}(x,y)$ was sampled on a regular 1024 by 1024 grid. The optimal bandwidth for a symmetric Gaussian kernel for KDE was calculated using Botev's algorithm [41]. Using a modified version of Botev's Matlab implementation of two-dimensional KDE, the same bandwidth was used for KDE of the random pattern in Ω_i_ yielding the CSR density function $\rho_{\mathrm{CSR}}(x,y)$.

## SUPPLEMENTARY MATERIAL FIGURES



## References

[1] Carmeliet P (2005). Angiogenesis in life, disease and medicine. Nature.

[2] Folkman J (1995). Angiogenesis in cancer, vascular, rheumatoid and other disease. Nat Med.

[3] Pusztaszeri MP, Seelentag W, Bosman FT (2006). Immunohistochemical expression of endothelial markers CD31, CD34, von Willebrand factor, and Fli-1 in normal human tissues. J Histochem Cytochem.

[4] Tomlinson J, Barsky SH, Nelson S, Singer S, Pezeshki B, Lee MC, Eilber F, Nguyen M (1999). Different patterns of angiogenesis in sarcomas and carcinomas. Clin Cancer Res.

[5] Eberhard A, Kahlert S, Goede V, Hemmerlein B, Plate KH, Augustin HG (2000). Heterogeneity of angiogenesis and blood vessel maturation in human tumors: implications for antiangiogenic tumor therapies. Cancer Res.

[6] Nico B, Benagiano V, Mangieri D, Maruotti N, Vacca A, Ribatti D (2008). Evaluation of microvascular density in tumors: pro and contra. Histol Histopathol.

[7] Sprindzuk M, Dmitruk A, Kovalev V, Bogush A, Tuzikov A, Liakhovski V, Fridman M (2009). Computer-aided Image Processing of Angiogenic Histological. J Clin Med Res.

[8] Weidner N, Folkman J, Pozza F, Bevilacqua P, Allred EN, Moore DH, Meli S, Gasparini G (1992). Tumor angiogenesis: a new significant and independent prognostic indicator in early-stage breast carcinoma. J Natl Cancer Inst.

[9] Murri AM, Hilmy M, Bell J, Wilson C, McNicol AM, Lannigan A, Doughty JC, McMillan DC (2008). The relationship between the systemic inflammatory response, tumour proliferative activity, T-lymphocytic and macrophage infiltration, microvessel density and survival in patients with primary operable breast cancer. Br J Cancer.

[10] Gasparini G, Weidner N, Bevilacqua P, Maluta S, Dalla Palma P, Caffo O, Barbareschi M, Boracchi P, Marubini E, Pozza F (1994). Tumor microvessel density, p53 expression, tumor size, and peritumoral lymphatic vessel invasion are relevant prognostic markers in node-negative breast carcinoma. J Clin Oncol.

[11] Mohammed ZM, McMillan DC, Edwards J, Mallon E, Doughty JC, Orange C, Going JJ (2013). The relationship between lymphovascular invasion and angiogenesis, hormone receptors, cell proliferation and survival in patients with primary operable invasive ductal breast cancer. BMC Clin Pathol.

[12] Des Guetz G, Uzzan B, Nicolas P, Cucherat M, Morere JF, Benamouzig R, Breau JL, Perret GY (2006). Microvessel density and VEGF expression are prognostic factors in colorectal cancer. Meta-analysis of the literature. Br J Cancer.

[13] Erbersdobler A, Isbarn H, Dix K, Steiner I, Schlomm T, Mirlacher M, Sauter G, Haese A (2010). Prognostic value of microvessel density in prostate cancer: a tissue microarray study. World J Urol.

[14] Concato J, Jain D, Li WW, Risch HA, Uchio EM, Wells CK (2007). Molecular markers and mortality in prostate cancer. BJU Int.

[15] Tretiakova M, Antic T, Binder D, Kocherginsky M, Liao C, Taxy JB, Oto A (2013). Microvessel density is not increased in prostate cancer: digital imaging of routine sections and tissue microarrays. Hum Pathol.

[16] Weidner N, Semple JP, Welch WR, Folkman J (1991). Tumor angiogenesis and metastasis—correlation in invasive breast carcinoma. N Engl J Med.

[17] Chalkley HW (1943). Method for the quantitative morphologic analysis of tissues. J Natl Cancer Inst.

[18] Hansen S, Sorensen FB, Vach W, Grabau DA, Bak M, Rose C (2004). Microvessel density compared with the Chalkley count in a prognostic study of angiogenesis in breast cancer patients. Histopathology.

[19] Vermeulen PB, Gasparini G, Fox SB, Colpaert C, Marson LP, Gion M, Belien JA, de Waal RM, Van Marck E, Magnani E, Weidner N, Harris AL, Dirix LY (2002). Second international consensus on the methodology and criteria of evaluation of angiogenesis quantification in solid human tumours. Eur J Cancer.

[20] Mikalsen LT, Dhakal HP, Bruland OS, Naume B, Borgen E, Nesland JM, Olsen DR (2013). The clinical impact of mean vessel size and solidity in breast carcinoma patients. PLoS One.

[21] West CC, Brown NJ, Mangham DC, Grimer RJ, Reed MW (2005). Microvessel density does not predict outcome in high grade soft tissue sarcoma. Eur J Surg Oncol.

[22] Belien JA, Somi S, de Jong JS, van Diest PJ, Baak JP (1999). Fully automated microvessel counting and hot spot selection by image processing of whole tumour sections in invasive breast cancer. J Clin Pathol.

[23] Bossi P, Viale G, Lee AK, Alfano R, Coggi G, Bosari S (1995). Angiogenesis in colorectal tumors: microvessel quantitation in adenomas and carcinomas with clinicopathological correlations. Cancer Res.

[24] Baddeley A, Turner R (2005). Spatstat: an R package for analyzing spatial point patterns. J Stat Soft.

[25] Chantrain CF, DeClerck YA, Groshen S, McNamara G (2003). Computerized quantification of tissue vascularization using high-resolution slide scanning of whole tumor sections. J Histochem Cytochem.

[26] van Niekerk CG, van der Laak JA, Borger ME, Huisman HJ, Witjes JA, Barentsz JO, Hulsbergen-van de Kaa CA (2009). Computerized whole slide quantification shows increased microvascular density in pT2 prostate cancer as compared to normal prostate tissue. Prostate.

[27] Borren A, Groenendaal G, Moman MR, Boeken Kruger AE, van Diest PJ, van Vulpen M, Philippens ME, van der Heide UA (2014). Accurate prostate tumour detection with multiparametric magnetic resonance imaging: dependence on histological properties. Acta Oncol.

[28] Sullivan CA, Ghosh S, Ocal IT, Camp RL, Rimm DL, Chung GG (2009). Microvessel area using automated image analysis is reproducible and is associated with prognosis in breast cancer. Hum Pathol.

[29] Balsat C, Blacher S, Signolle N, Beliard A, Munaut C, Goffin F, Noel A, Foidart JM, Kridelka F (2011). Whole slide quantification of stromal lymphatic vessel distribution and peritumoral lymphatic vessel density in early invasive cervical cancer: a method description. ISRN Obstet Gynecol.

[30] Caie PD, Turnbull AK, Farrington SM, Oniscu A, Harrison DJ (2014). Quantification of tumour budding, lymphatic vessel density and invasion through image analysis in colorectal cancer. J Transl Med.

[31] Angell HK, Gray N, Womack C, Pritchard DI, Wilkinson RW, Cumberbatch M (2013). Digital pattern recognition-based image analysis quantifies immune infiltrates in distinct tissue regions of colorectal cancer and identifies a metastatic phenotype. Br J Cancer.

[32] Naschberger E, Croner RS, Merkel S, Dimmler A, Tripal P, Amann KU, Kremmer E, Brueckl WM, Papadopoulos T, Hohenadl C, Hohenberger W, Sturzl M (2008). Angiostatic immune reaction in colorectal carcinoma: Impact on survival and perspectives for antiangiogenic therapy. Int J Cancer.

[33] Gerlinger M, Rowan AJ, Horswell S, Larkin J, Endesfelder D, Gronroos E, Martinez P, Matthews N, Stewart A, Tarpey P, Varela I, Phillimore B, Begum S (2012). Intratumor heterogeneity and branched evolution revealed by multiregion sequencing. N Engl J Med.

[34] Hanahan D, Weinberg RA (2011). Hallmarks of cancer: the next generation. Cell.

[35] Floor SL, Dumont JE, Maenhaut C, Raspe E (2012). Hallmarks of cancer: of all cancer cells, all the time?. Trends Mol Med.

[36] Diaz-Cano SJ (2008). General morphological and biological features of neoplasms: integration of molecular findings. Histopathology.

[37] Owen MR, Alarcon T, Maini PK, Byrne HM (2009). Angiogenesis and vascular remodelling in normal and cancerous tissues. J Math Biol.

[38] Minchinton AI, Tannock IF (2006). Drug penetration in solid tumours. Nat Rev Cancer.

[39] Rajaganeshan R, Prasad R, Guillou PJ, Chalmers CR, Scott N, Sarkar R, Poston G, Jayne DG (2007). The influence of invasive growth pattern and microvessel density on prognosis in colorectal cancer and colorectal liver metastases. Br J Cancer.

[40] Fuchs TJ, Buhmann JM (2011). Computational pathology: challenges and promises for tissue analysis. Comput Med Imaging Graph.

[41] Botev ZI, Grotowski JF, Kroese DP (2010). Kernel density estimation via diffusion. Ann Stat.

[42] Lopez XM, Debeir O, Maris C, Rorive S, Roland I, Saerens M, Salmon I, Decaestecker C (2012). Clustering methods applied in the detection of Ki67 hot-spots in whole tumor slide images: an efficient way to characterize heterogeneous tissue-based biomarkers. Cytometry A.

[43] Song Y, Treanor D, Bulpitt AJ, Magee DR (2013). 3D reconstruction of multiple stained histology images. J Pathol Inform.

[44] Gottwald E, Kleintschek T, Giselbrecht S, Truckenmuller R, Altmann B, Worgull M, Dopfert J, Schad L, Heilmann M (2013). Characterization of a chip-based bioreactor for three-dimensional cell cultivation via Magnetic Resonance Imaging. Z Med Phys.

[45] Cromey DW (2010). Avoiding twisted pixels: ethical guidelines for the appropriate use and manipulation of scientific digital images. Sci Eng Ethics.

[46] Reyes-Aldasoro CC, Williams LJ, Akerman S, Kanthou C, Tozer GM (2011). An automatic algorithm for the segmentation and morphological analysis of microvessels in immunostained histological tumour sections. J Microsc.

[47] Reyes-Aldasoro CC, Bjorndahl MA, Akerman S, Ibrahim J, Griffiths MK, Tozer GM (2012). Online chromatic and scale-space microvessel-tracing analysis for transmitted light optical images. Microvasc Res.

[48] Ruifrok AC, Johnston DA (2001). Quantification of histochemical staining by color deconvolution. Anal Quant Cytol Histol.

[49] Yen JC, Chang FJ, Chang S (1995). A new criterion for automatic multilevel thresholding. IEEE Trans Image Process.

[50] Martinez K, Cupitt J (2005). VIPS ― a highly tuned image processing software architecture. IEEE ICIP.

[51] Walter T, Shattuck DW, Baldock R, Bastin ME, Carpenter AE, Duce S, Ellenberg J, Fraser A, Hamilton N, Pieper S, Ragan MA, Schneider JE, Tomancak P (2010). Visualization of image data from cells to organisms. Nat Methods.

