# Supplementary material for: Continuous representation of tumor microvessel density and detection of angiogenic hotspots in histological whole-slide images
Source: Oncotarget. 2015 Jun 8;6(22):19163–76. doi: 10.18632/oncotarget.4383 (PMC4662482; doi:10.18632/oncotarget.4383)
Supplement: Supplementary file 1 [file oncotarget-06-19163-s001.pdf]

Continuous representation of tumor microvessel density and detection of angiogenic hotspots in histological whole-slide images

Supplementary Material

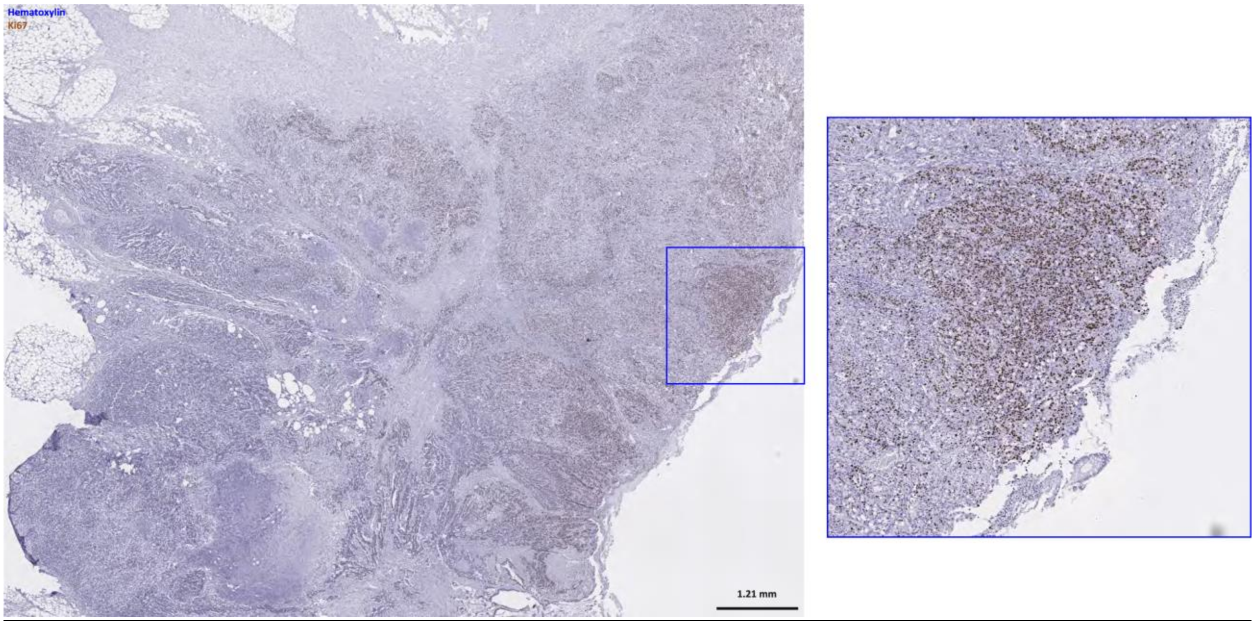

Suppl. Fig. 1: Tumor cell proliferation compared to blood microvessel density

Ki67 staining of the colon tumor sample that was used for Fig. 2A (CD34 staining overview) and Fig. 4 (hotspot detection in this sample). In the present figure, it can be appreciated that Ki67-positive proliferating cells are present in the tumor. Ki67-positivity is most pronounced at the right-hand tumor margin. This pronounced tumor cell proliferation spatially corresponds to the location of tumor angiogenic hotspots in the same sample as shown in Fig. 4C.

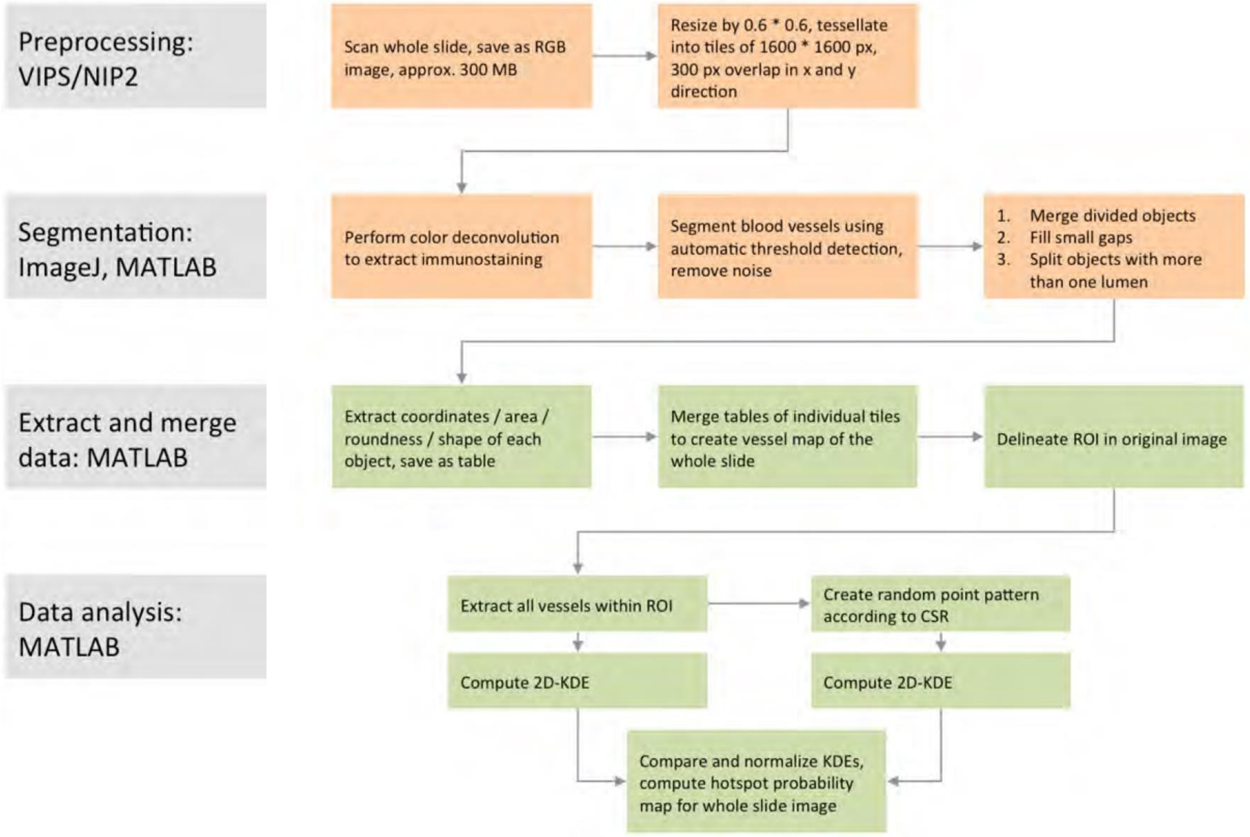

Suppl. Fig. 2: Computational procedures flowchart

A flowchart of the computational procedures. Orange: Steps that involve manipulation of image data. These steps are computationally intensive. Green: Steps that involve manipulation of vessel coordinate datasets. These steps are computationally inexpensive because the data has been reduced to a table where each entry corresponds to one tumor vessel. KDE = kernel density estimation, CSR = complete spatial randomness.

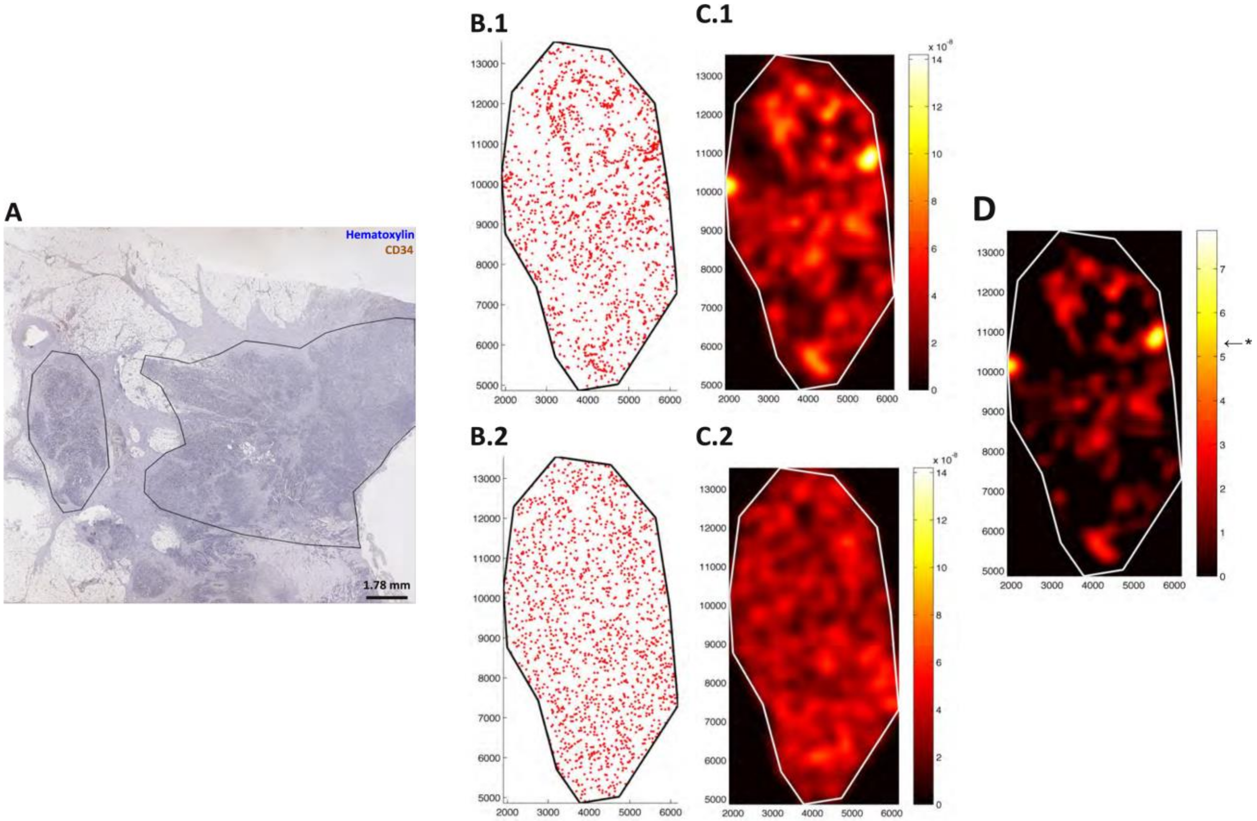

Supplemental Fig. 3: Hematoxylin/DAB immunostaining for colorectal tumor sample 0, region 2

Complementing Figs. 2 and 4 in the main manuscript, this figure shows tumor vessel map (B1: observed, B2: random), vessel density function (C1: observed, C2: random) and hotspot probability map (D) for the left-hand region in Fig. 2A. C1, C2: color map represents arbitrary units; D: color map represents standard deviations of the random pattern. KDE bandwidth for C: 153 px. Bonferroni-corrected level of significance in D is 5.28 standard deviations (marked by *). Two contiguous areas (yellow) fulfill this criterion for an angiogenic hotspot.

Details of the computational procedures

- Microscope camera “Aperio ScanScope”: Magnification 20x, images saved as SVS files
- Image preprocessing in VIPS/Nip2: Downscale by factor 0.6 in each dimension, tessellation yields tiles of 1600x1600 px size with 300x300 px overlap, save tiles as PNG (lossless compression, level 1 of 9, approx. 4 MB per tile); full whole slide image saved as JPG compressed TIFF file for ROI delineation (lossy compression, level 70%, no interlace). Tile overlap and subsequent reconstruction ensured that no boundary artifacts occured.
- ImageJ/Fiji color deconvolution and thresholding: Minimal particle size 8 px
- MATLAB program: minimal hole size = 11 px, minimal object size for morphological post-processing = 17 px, final minimal object size = 65 px
- All computational analyses were performed on a standard desktop workstation (16 GB DDR3 RAM, 2.2 GHz Intel® Core™ i7 processor, Intel® Iris Pro™ Graphics).
- Computationally intensive image analysis steps (orange boxes in Suppl. Fig. 1) typically took several hours per whole slide image while data analysis of the vessel maps typically took less than one minute. No systematic attempt to make the code faster was made.
